# The Short-Term Change in Knowledge of Cannabis-Related Risks After a Brief Curriculum-Integrated School Intervention Among Adolescents: A Quasi-Experimental Pre–Post Study

**DOI:** 10.3390/healthcare14101264

**Published:** 2026-05-07

**Authors:** José Carlos Azón-Belarre, Patricia Berges-Usán, Piedad Gómez-Torres, Jessica Romeo-García, María Teresa García-Guerra, María José Membrive-Jiménez, Sergio Galarreta-Aperte

**Affiliations:** 1 Faculty of Health Sciences, San Jorge University, Villanueva de Gállego, 50830 Zaragoza, Spain; jcazon@usj.es (J.C.A.-B.); mtgarcia@usj.es (M.T.G.-G.); 2 Cinco Villas Psychosocial Rehabilitation Center, 50600 Ejea de los Caballeros, Spain; pberges@iniciativasocial.org; 3 Department of Nursing, Faculty of Health Sciences of Ceuta, University of Granada, 51001 Ceuta, Spain; mjmembrive@ugr.es (M.J.M.-J.); sgalarreta@ugr.es (S.G.-A.); 4 Miguel Servet University Hospital, 50008 Zaragoza, Spain; jessiromeogarcia@gmail.com; 5 Pere Virgili Health Research Institute, 43007 Reus, Spain

**Keywords:** adolescents, cannabis, school-based intervention, substance use prevention, health education, health literacy, program evaluation

## Abstract

**Highlights:**

**What are the main findings?**

- A brief nurse-led (3 h) cannabis education program was associated with short-term improvement in cannabis-related knowledge among Spanish secondary students.
- The median knowledge score increased from 8 to 13/15 at two weeks, with improvement observed in 96.7% of participants (Wilcoxon r = 0.867).

**What are the implications of the main findings?**

- This study provides a pragmatic estimate of the short-term change in a proximal cognitive outcome under routine school conditions.
- Controlled studies with longer follow-up and implementation measures are needed before drawing conclusions about effectiveness, sustained impact, feasibility, or scalability.

**Abstract:**

**Purpose:** The purpose of this study was to estimate the short-term pre–post change in cannabis risk knowledge after a brief curriculum-integrated school intervention delivered under routine educational conditions. **Methods:** This was a quasi-experimental single-group pre–post study without a control group in a secondary education setting (*n* = 151; ages 15–17). Intervention: Three standardized 1 h classroom sessions delivered by health professionals, focused on health literacy, risk perception, myth debunking, and peer pressure. Primary outcome: Pre–post change in a knowledge scale (0–15). Analysis: Wilcoxon signed-rank test; effect size r. **Results:** Knowledge scores increased from a median of 8 (IQR 7–10) at pretest to 13 (IQR 12–14.5) at posttest. The median paired change was +5 points (IQR 3–6; Hodges–Lehmann estimate 5.0, 95% CI 4.5 to 5.0), with improvement observed in 96.7% of students (146/151); the effect size was large (*r* = 0.867). Associations with family and clinical variables were considered exploratory due to low subgroup frequencies. **Discussion:** In this single-group pre–post study, the intervention was associated with short-term improvement in cannabis risk knowledge. These findings should be interpreted as evidence of short-term within-subject change in a proximal cognitive outcome under uncontrolled conditions, not as evidence of intervention effectiveness, sustained learning, behavioral change, or broader practical impact. **Conclusions:** Brief curriculum-integrated cannabis education may be a feasible format for generating short-term gains in risk knowledge, but controlled studies with longer follow-up, psychometrically stronger outcome measures, and implementation data are needed to assess the durability and behavioral relevance of these changes.

## 1. Introduction

Cannabis is the most widely used illicit substance among adolescents and young adults in many Western contexts [1,2,3]. Use occurs amid increasing product diversity and THC potency [2,4], which may heighten risks during adolescence, a period of vulnerable neurocognitive development [5,6]. European school-survey data indicate substantial recent use among secondary school students, supporting early school-based prevention [1]. Cannabis-related knowledge and perceived risk are modifiable targets associated with lower use and intention to use [7]. In addition, adolescents’ cannabis-related beliefs are increasingly shaped by online environments, and recent evidence points to both associations between social media exposure and more favorable cannabis attitudes and persistent knowledge gaps regarding the risks and perceived benefits of different cannabis products [8,9]. Adolescent use is linked to poorer academic outcomes, mental health problems, and the risk of cannabis use disorder [10,11], potentially amplified by higher-potency products. In Spain, cannabis remains the most prevalent illicit substance among secondary school students. According to ESTUDES 2023, 26.9% of students aged 14–18 years reported lifetime use, 21.8% use in the past 12 months, and 15.6% use in the past 30 days, underscoring its relevance as a public health issue in this age group [12].

Although the prevention literature is extensive, much evidence comes from multicomponent or controlled school-based programs [13,14]. However, translating evidence-based prevention into routine school practice remains challenging, because program outcomes depend not only on intervention content but also on adoption, fidelity, delivery conditions, and implementation context [15]. Time, resources, and curricular demands often hinder the implementation of lengthy programs, creating a gap between experimental evidence and what is feasible in routine school conditions [16,17]. Brief curriculum-integrated interventions should therefore be evaluated under usual conditions, with the explicit reporting of feasibility and the magnitude of short-term change in early cognitive mediators [16,18,19,20]. Estimating the change achievable with brief standardized formats may inform implementation decisions across settings.

Recent school-based approaches using digital delivery, harm reduction framing, or drug literacy components have reported improvements in drug literacy and risk perception and, in some trials, slower uptake of risky use, suggesting relevant directions for future school-based prevention research [21,22,23].

Pragmatic evaluations quantifying the cognitive change achievable with very brief standardized curriculum-based interventions under usual school conditions remain scarce [17]. Quantifying change with a limited total dose can provide a benchmark for selecting feasible interventions and designing subsequent controlled evaluations.

Within this framework, we implemented “5 Top Secrets about Cannabis,” a brief curriculum-integrated school intervention designed to be deliverable under usual educational conditions. The primary objective was to estimate the magnitude of the short-term change in specific knowledge about cannabis-related risks achievable with a brief standardized curricular intervention using a pre–post design. Secondary objectives were to describe the self-reported prevalence of cannabis use and other addictive behaviors to contextualize the sample and to explore associations with selected family and contextual factors on a strictly exploratory basis and without inferential intent. We hypothesized that cannabis risk knowledge (0–15 score) would significantly increase from pretest to posttest.

## 2. Materials and Methods

### 2.1. Study Design

We conducted a quasi-experimental pre–post study without a control group to estimate short-term change in knowledge about cannabis-related risks following a brief school-based intervention. This manuscript follows the TREND Statement Checklist (Supplementary Material S1) [24].

### 2.2. Settings and Participants

This study was conducted in three secondary schools in a municipality in Aragon (Spain) with a mixed urban–rural catchment area. Schools were mainstream secondary education settings, and the intervention was delivered during regular class time. Eligible participants were students in the 3rd and 4th years of compulsory secondary education who were present at baseline. Families received written information through the school’s usual channel; legal guardians provided written consent, and students provided classroom assent. The total number of eligible students who received the invitation and reasons for non-participation were not systematically recorded; therefore, the participation rate could not be calculated. We excluded students without consent or with >60% missing items on the primary outcome.

### 2.3. Intervention

The “5 Top Secrets about Cannabis” intervention was integrated into regular class time and delivered by mental health specialist nurses in three 1 h classroom sessions over three consecutive weeks. The program emphasized health literacy, the correction of misconceptions, risk perception, peer influence, and resistance skills, using evidence-based information on potency/risks and peer pressure scenarios. Content and delivery were standardized via a common script and uniform materials. Sessions were delivered by health professionals external to the teaching staff; no screening or clinical activities were conducted. Attendance was recorded and was complete.

To support replication, the intervention was standardized using a facilitator script and uniform materials (three 1 h sessions; core messages on cannabis-related health risks, potency/product diversity, and peer pressure resistance).

### 2.4. Data Collection Procedures

An anonymous self-administered questionnaire (Supplementary Material S2) was completed at two time points, immediately before the first session (pretest) and two weeks after the final session (approximately five weeks after baseline–posttest), during school hours. Students generated an alphanumeric code to match questionnaires across waves without collecting identifying information. Questionnaires with >60% missing items on the primary outcome were excluded; missing data were not imputed, and pre–post analyses used complete pairs.

All student-facing materials were administered in Spanish; any English versions provided for transparency are translations of the original materials.

### 2.5. Variables and Measures

Primary outcome: Cannabis knowledge score (0–15), calculated as the sum of 15 true/false/don’t know items (1 point per correct response; higher scores indicate greater knowledge). The scale was developed to assess the acquisition of the intervention’s core messages and common adolescent myths, including health/safety content and a small number of context-specific items on traffic/administrative consequences. It was designed for short-term sensitivity rather than cross-study comparability. Responses were true/false/don’t know; “don’t know” responses were scored as incorrect. Because the scale was developed specifically for this intervention and closely reflected its core content, it was intended to maximize short-term sensitivity rather than provide a standardized or broadly validated measure. No formal psychometric evaluation (e.g., reliability, construct validity, or responsiveness beyond this context) was conducted prior to this study.

Secondary outcomes: Pre–post change in knowledge score (Δ = post − pre); use (daily/non-daily/none) of cannabis, alcohol, amphetamines, cocaine, and gambling; contextual and family factors, consisting of sex, age, residence (urban/rural), current follow-up in mental health services (yes/no), and parental use (father/mother). Parental substance use (father and mother) was assessed via adolescent report, separately for each parent and for each substance. For alcohol and cannabis, use was coded as at least monthly (≥1 time per month) versus no use. In this sample, reported parental use fell exclusively into three categories (alcohol, cannabis, or no drugs). All substance uses and gambling variables referred to the past 3 months; non-daily cannabis use was defined as reporting monthly use (1–4 times per month) during this period and “daily” as everyday use.

For exploratory analyses, a change score was defined as posttest minus pretest knowledge score. For exploratory subgroup analyses, a binary variable indicating any reported parental alcohol or cannabis use (yes/no) was derived from adolescent report.

### 2.6. Sample Size

No a priori sample size calculation was performed because this study was conceived as an implementation evaluation under routine educational conditions. The sample depended on the accessible population and attendance on the day of the pretest. We report descriptive precision and effect size estimates to contextualize practical relevance. This study was not designed for population inference or to detect a prespecified minimum effect size but to estimate the empirical magnitude of short-term cognitive change under usual school conditions.

### 2.7. Statistical Analysis

Analyses were conducted in R software (R Foundation for Statistical Computing, Vienna, Austria). Categorical variables are reported as n (%) and continuous variables as the median (IQR). Pre–post comparisons used the Wilcoxon signed-rank test; subgroup comparisons used nonparametric tests as appropriate; and associations between categorical variables used Fisher’s exact test. Effect size was calculated as Wilcoxon r (Z/√N). Secondary analyses were exploratory, and no adjustment for multiple comparisons was applied.

Exploratory subgroup analyses. In response to peer review comments, we conducted exploratory subgroup analyses of the primary outcome. Within each subgroup, the pre–post change in the knowledge score was assessed using the Wilcoxon signed-rank test and summarized as pre- and post-intervention medians (IQR), median change (IQR), proportion improved, and effect size *r*. To explore whether the magnitude of change differed across subgroups, change scores (post − pre) were compared using the Mann–Whitney U test for binary variables and the Kruskal–Wallis test for variables with more than two categories. These analyses were exploratory, were not adjusted for multiple comparisons, and should not be interpreted inferentially, particularly in subgroups with very small cell sizes.

### 2.8. Ethical Considerations

This study was approved by the Ethics Committee of San Jorge University (PI-13/1/25-26). This study was conducted in accordance with the Declaration of Helsinki. Participation was voluntary; written informed consent was obtained from legal guardians and anonymity was guaranteed.

## 3. Results

### 3.1. Sample Characteristics

The total number of eligible students invited and the reasons for non-participation were not systematically recorded. A total of 151 students provided guardian consent and student assent, were enrolled, and received the intervention. All enrolled participants completed both assessments (pretest *n* = 151; posttest *n* = 151; retention 100%). No questionnaires met the exclusion criterion of >60% missing items on the primary outcome (excluded *n* = 0). No protocol deviations were recorded. Of the 151 participants, 77 were female (51.0%), and 117 lived in an urban area (77.4%). Age ranged from 15 to 17 years (median 16).

### 3.2. Pre–Post Change in Knowledge

**Figure 1:**
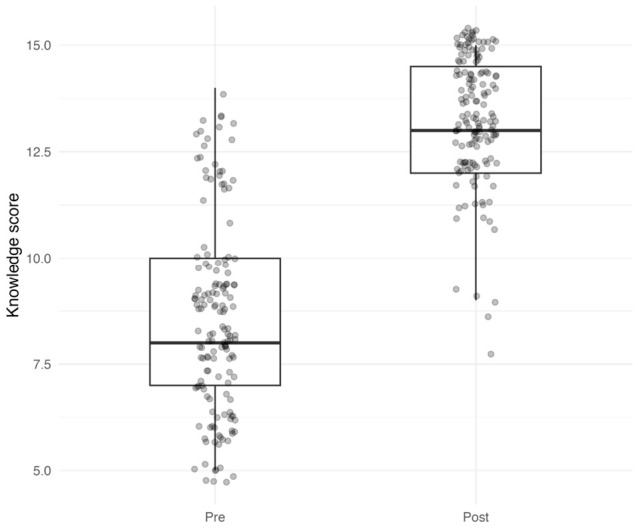
Pre- and post-intervention knowledge scores in the full sample. Boxes indicate the median and interquartile range; points represent individual participants.

Knowledge scores increased from a median of 8 (IQR 7–10) at pretest to 13 (IQR 12–14.5) at posttest (Figure 1). The change was statistically significant (Wilcoxon signed-rank *p* < 0.001), with a median paired change of +5 points (IQR 3–6; Hodges–Lehmann estimate 5.0, 95% CI 4.5 to 5.0). Improvement was observed in 146 students (96.7%), and the effect size was large (*r* = 0.867). No adverse events or unintended effects were reported during assessment.

### 3.3. Exploratory Subgroup Analyses of Pre–Post Change in Knowledge

Pre–post improvement in knowledge scores was observed across cannabis use strata and in students with and without any reported parental alcohol/cannabis use (Table 1).

**Figure 2:**
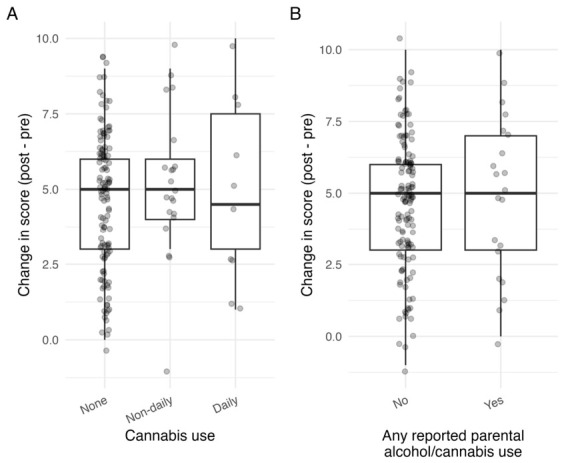
(**A**) The change in the knowledge score (post − pre) according to cannabis use status. Boxes indicate the median and interquartile range; points represent individual participants. (**B**) The change in the knowledge score (post − pre) according to any reported parental alcohol/cannabis use. Boxes indicate the median and interquartile range; points represent individual participants.

The median change was +5 points among non-users, +5 among non-daily cannabis users, and +4.5 among daily users. The median change was also +5 points in students both with and without any reported parental alcohol/cannabis use. However, change scores did not differ significantly by cannabis use status (Kruskal–Wallis *p* = 0.474; Figure 2A) or by any reported parental alcohol/cannabis use (Mann–Whitney *p* = 0.684; Figure 2B). Additional exploratory subgroup analyses by alcohol use, amphetamine use, cocaine use, and gambling are presented in Supplementary Table S1. These results should be interpreted with extreme caution because several subgroups were very small. In particular, the apparent difference by alcohol use was based on only four students in the no-alcohol subgroup, and inferential statistics were not interpreted for the cocaine use subgroup because only one participant reported non-daily use.

### 3.4. Prevalence of Substance Use and Contextual Characteristics

Self-reported prevalence is shown in Supplementary Figure S1. Most students reported no cannabis use (79.5%); 13.9% reported non-daily use and 6.6% daily use. Alcohol use was mostly non-daily (97.4%), with no daily use. No differences by sex or residence were observed for the use of any substance (all *p* > 0.20).

Exploratory associations between substance use and selected contextual factors are presented in Supplementary Table S2. An association was observed between mental health follow-up and non-daily amphetamine use (*p* = 0.032). Non-daily amphetamine use was reported by 16.6% (2/12) of students with mental health follow-up compared with 1.5% (2/139) of those without follow-up. Exploratory associations between adolescents’ substance use patterns and reported parental substance use are presented in Supplementary Table S3. Reported paternal use was associated with student cannabis use (*p* = 0.024) and amphetamine use (*p* < 0.001), whereas no significant associations were observed for reported maternal use. Any cannabis use (non-daily or daily) was reported by 40% (2/5) of students reporting paternal alcohol use and 40% (4/10) reporting paternal cannabis use, versus 18.4% (25/136) among those reporting no paternal use. Given the very small cell frequencies, these analyses should be interpreted as exploratory and hypothesis-generating only.

Pre–post change was assessed with the Wilcoxon signed-rank test within each subgroup. Between-group comparisons of change scores (post − pre) were assessed using the Kruskal–Wallis test for cannabis use and the Mann–Whitney U test for reported parental alcohol/cannabis use. These analyses were exploratory and should not be interpreted inferentially. Reported parental alcohol/cannabis use was defined as any adolescent-reported alcohol or cannabis use by either parent (yes/no).

## 4. Discussion

In routine school settings, this brief curriculum-integrated intervention was associated with a short-term increase in a content-specific measure of cannabis risk knowledge assessed two weeks after the final session. Because this was a single-group pre–post study, the observed change cannot be attributed exclusively to the intervention and may also reflect testing effects, short-term recall, item familiarization, maturation, or other uncontrolled influences. Accordingly, the findings should be interpreted as documenting short-term within-sample change in a proximal cognitive outcome, rather than as evidence of intervention effectiveness or superiority over other preventive approaches. This interpretation is consistent with reviews showing that universal school-based prevention programs often have small or null effects on cannabis use, whereas interactive and higher-dose skills-based programs tend to show more consistent benefits [13,20,25,26,27,28].

The main contribution of this study is to quantify, under routine school implementation and with a total dose of three hours, the magnitude of short-term cognitive change in specific cannabis-related knowledge. This provides a pragmatic reference point for future controlled and implementation-focused evaluations of brief school-based prevention formats.

The magnitude of change should be interpreted in light of the assessment timing and the type of outcome measured. Because the posttest was administered two weeks after the final session, the findings likely reflect short-term recall or near-term retention of intervention content rather than sustained knowledge consolidation. In addition, the primary outcome was measured using an ad hoc scale developed to reflect the intervention’s specific content. Although this may increase sensitivity to detect near-term change, it limits comparability with other studies and raises the possibility of content alignment or teaching-to-test effects. Because no psychometric evaluation was performed, the reliability, validity, interpretability, and responsiveness of the measure remain uncertain. A recent systematic review in adolescents indicates that higher cannabis-related knowledge and higher perceived risk are often associated with lower current use and lower intentions to use, supporting these constructs as proximal prevention targets [7]. Therefore, the observed change should be interpreted as an intermediate cognitive outcome only, without conclusions about prevention impact, behavioral change, or durability.

Importantly, longitudinal research suggests that perceived risk is not only a predictor of later cannabis use but also a consequence of use: initiation and continued use can lead to decreases in perceived risk over time, reinforcing the need for repeated messaging and components that target normative beliefs and risk appraisal. In Spain, national survey analyses similarly show that lower perceived risk and greater perceived accessibility are associated with a higher likelihood of cannabis use among adolescents, indicating that prevention content may benefit from explicitly addressing both risk perception and perceived availability [29,30]. Educational content also needs to keep pace with the rapidly changing cannabis landscape in Europe. Recent monitoring describes increases in THC potency, diversification of consumer products (e.g., vaping products, edibles, and high-potency concentrates), and the emergence of semi-synthetic cannabinoids, all of which can complicate adolescents’ understanding of dose, routes of administration, and risk. This supports the value of curricula that move beyond “cannabis” as a single product category and include product- and harm-relevant information aligned with current epidemiological and market indicators [31]. In practice, these findings support considering brief curricular interventions as pragmatic approaches that warrant a further evaluation of early cognitive mediators, although studies with comparator groups and follow-up are required to assess the persistence of change and its relationship to behavioral outcomes [3,18].

Secondary analyses were intended only to describe the sample context and explore possible associations with selected family and clinical factors. Given the small cell frequencies in several categories, these findings should be interpreted exclusively as exploratory and hypothesis-generating. In particular, the observed relationships between reported parental use and student cannabis and amphetamine use, as well as the association between mental health follow-up and non-daily amphetamine use, should not be given inferential weight. Nevertheless, the patterns observed for parental cannabis use are consistent with previous evidence linking parental cannabis use with a higher likelihood of cannabis initiation and use among offspring [32,33].

The strengths of this study include the evaluation of a brief preventive intervention delivered under routine school conditions, integrated into the curriculum and implemented in three schools using standardized classroom sessions led by health professionals. The paired pre–post design allowed for the estimation of short-term change in a relevant cognitive outcome in an adolescent sample. However, several limitations constrain interpretation. First, the absence of a control group prevents attributing the observed change exclusively to the intervention and does not allow alternative explanations, such as history, maturation, testing effects, or item familiarization, to be ruled out. Second, implementation in a single municipality limits the direct generalizability of the effect magnitude. Third, because the total number of eligible students invited and the reasons for non-participation were not systematically recorded, the participation rate could not be estimated, and potential selection bias cannot be excluded. Fourth, evaluation focused on a short-term cognitive outcome without longitudinal follow-up or the measurement of behavioral change. Finally, although the intervention was standardized through a common script and materials, formal implementation measures such as fidelity, adherence, quality of delivery, and participant engagement were not collected; therefore, the consistency of delivery cannot be verified, and feasibility or scalability should not be inferred from these data alone. Secondary analyses should also be considered strictly exploratory because of the limited sample size and small subgroup frequencies.

Future studies should use controlled designs, longer follow-up, and standardized or psychometrically evaluated outcome measures to assess whether short-term changes in knowledge are sustained and whether they are associated with trajectories of use during adolescence. Process and implementation measures, including fidelity, received dose, and participant engagement, would also strengthen interpretation under routine conditions [15,28,34,35]. Recent school-based approaches using digital delivery, harm reduction framing, or drug literacy components may offer useful directions for future evaluation [21,22,23].

## 5. Conclusions

A brief curriculum-integrated school intervention delivered under routine conditions was associated with short-term improvement in a content-specific measure of cannabis risk knowledge among secondary school adolescents. These findings should be interpreted as evidence of short-term change in a proximal cognitive outcome under uncontrolled conditions, rather than as evidence of intervention effectiveness, sustained learning, or behavioral impact. Future controlled studies with longer follow-up, psychometrically stronger outcome measures, and implementation data are needed to assess the persistence, interpretability, and potential behavioral relevance of these changes.

## Figures and Tables

**Table 1:** Exploratory pre–post change in cannabis risk knowledge overall and by selected subgroups.

Variable	Group	n	Pre Median (IQR)	Post Median (IQR)	Change Median (IQR)	Improved n (%)	Within-Group *p*	r
Overall	All participants	151	8 (7–10)	13 (12–14.5)	5 (3–6)	146 (96.7)	<0.001	0.867
Cannabis use	None	120	8 (7–10)	13 (12–14)	5 (3–6)	116 (96.7)	<0.001	0.868
	Non-daily	21	8 (7–9)	13 (13–15)	5 (4–6)	20 (95.2)	<0.001	0.868
	Daily	10	8 (6.25–9)	13 (13–14)	4.5 (3–7.5)	10 (100)	0.006	0.886
Reported parental alcohol/cannabis use	No	130	8 (7–10)	13 (12–14)	5 (3–6)	126 (96.9)	<0.001	0.866
	Yes	21	8 (7–9)	14 (13–15)	5 (3–7)	20 (95.2)	<0.001	0.877

Any reported parental alcohol/cannabis use was defined as any adolescent-reported alcohol or cannabis use by either parent (yes/no).

## Data Availability

The data presented in this study are available on request from the authors. The data are not publicly available due to privacy and ethical restrictions related to research with minors and sensitive substance-use information.

## Supplementary Materials

The following supporting information can be downloaded at https://www.mdpi.com/article/10.3390/healthcare14101264/s1, Supplementary Material S1: TREND [24]; Supplementary material S2: The 5 Top Secrets about Cannabis knowledge questionnaire; Supplementary Material S3: Supplementary Figure S1. Frequency of use by substance (past 3 months) among enrolled students (*n* = 151); Table S1. Exploratory pre–post change in cannabis risk knowledge in additional subgroups; Table S2. Substance use by contextual factors; Table S3. Associations between adolescents’ substance use and reported parental substance use.

## Abbreviations

The following abbreviations are used in this manuscript:

IQR	Interquartile Range
THC	Tetrahydrocannabinol
TREND	Transparent Reporting of Evaluations with Nonrandomized Designs
