# Supplementary material for: The Short-Term Change in Knowledge of Cannabis-Related Risks After a Brief Curriculum-Integrated School Intervention Among Adolescents: A Quasi-Experimental Pre–Post Study
Source: Healthcare (Basel). 2026 May 7;14(10):1264. doi: 10.3390/healthcare14101264 (PMC13206630; doi:10.3390/healthcare14101264)
Supplement: Supplementary file 1 [file healthcare-14-01264-s001.zip › Supplementary material S2. Knowledge questionaire.pdf]

Supplementary material S2.

5 Top Secrets about Cannabis knowledge questionnaire

Participants completed a cannabis knowledge questionnaire (true/false/don't know items) referring to their current knowledge at the time of assessment.

1. Cannabis use only affects people who use it often: True (T) False (F) DK
2. Most adolescents and adults smoke joints: True (T) False (F) DK

   You can be fined for smoking joints in the street: True (T) False (F) DK
3. Marijuana and hashish are the same substance: True (T) False (F) DK
4. Cannabis use increases appetite: True (T) False (F) DK
5. Smoking joints is healthier than smoking tobacco: True (T) False (F) DK
6. Cannabis contains a high percentage of adulterants: True (T) False (F) DK
7. Smoking joints does not reduce attention: True (T) False (F) DK
8. Joints can cure some diseases such as cancer: True (T) False (F) DK
9. Smoking joints can help solve problems: True (T) False (F) DK
10. Driving after using cannabis can result in the loss of points from your driver's license: True (T) False (F) DK
11. Joints always produce the same effects: True (T) False (F) DK
12. I can carry any amount of cannabis on me without legal consequences: True (T) False (F) DK
13. "Weed" reduces the harmful effects of tobacco: True (T) False (F) DK

14. When cannabis is used on medical advice, it is also used by smoking it:

True (T) False (F) DK
