# Supplementary material for: The Short-Term Change in Knowledge of Cannabis-Related Risks After a Brief Curriculum-Integrated School Intervention Among Adolescents: A Quasi-Experimental Pre–Post Study
Source: Healthcare (Basel). 2026 May 7;14(10):1264. doi: 10.3390/healthcare14101264 (PMC13206630; doi:10.3390/healthcare14101264)
Supplement: Supplementary file 1 [file healthcare-14-01264-s001.zip › Supplementary material S3. Figure 1. Tables S1, S2 and S3..pdf]

Supplementary material S3

Figure S1. Frequency of use by substance (past 3 months) among enrolled students (n = 151).

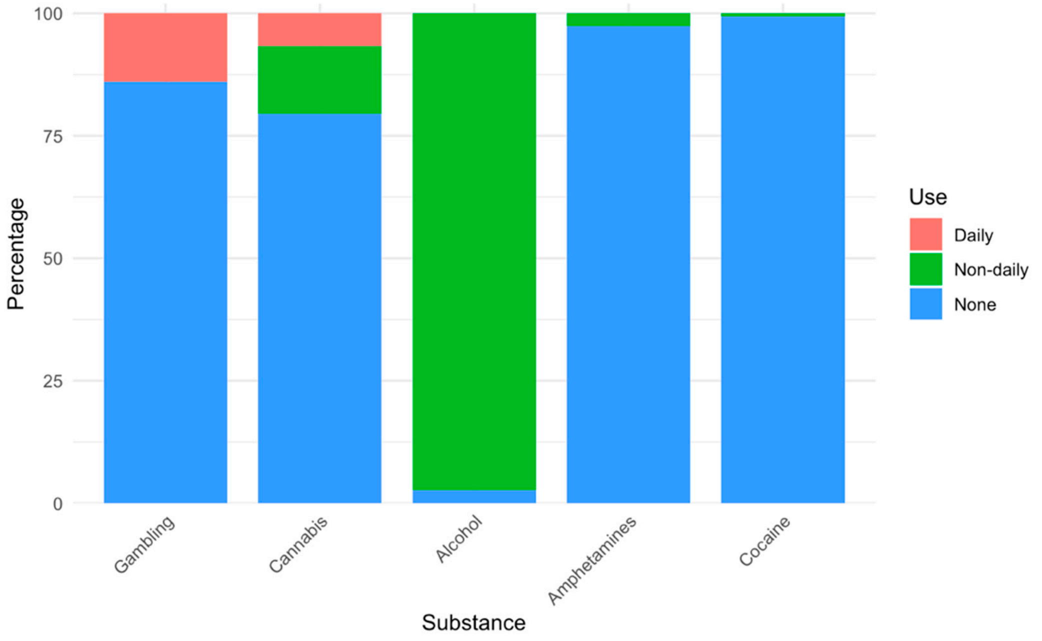

Table S1. Exploratory pre–post change in cannabis-risk knowledge in additional subgroups

Variable	Group	n	Pre median (IQR)	Post median (IQR)	Change median (IQR)	Improved n (%)	Within-group p	r
Alcohol use	None	4	10 (9–11.25)	12 (12–12.5)	2 (0.75–3.5)	3 (75.0)	.181	.926
	Non-daily	147	8 (7–10)	13 (13–15)	5 (3–6)	143 (97.3)	< .001	.866
Amphetamines use	None	147	8 (7–10)	13 (12–14)	5 (3–6)	142 (96.6)	< .001	.866
	Non-daily	4	7 (5.75–9)	14.5 (13.75–15)	6.5 (4.5–8.5)	4 (100.0)	.100	.913
Cocaine use	None	150	8 (7–10)	13 (12–14)	5 (3–6)	145 (96.7)	< .001	.866
	Non-daily	1	5 (5–5)	15 (15–15)	10 (10–10)	1 (100.0)	NA	NA
Gambling	None	130	8 (7–10)	13 (13–15)	5 (3–6)	127 (97.7)	< .001	.866
	Daily	21	9 (7–10)	13 (12–14)	4 (3–6)	19 (90.5)	< .001	.877

Within-group pre–post change was assessed with the Wilcoxon signed-rank test. Between-group comparisons of change scores (post – pre) were assessed with the Mann–Whitney U test. These analyses are reported for descriptive completeness only and should be interpreted with extreme

caution because several subgroups were very small. Inferential statistics were not interpreted for the cocaine-use subgroup because only one participant reported non-daily use.

Table S2. Substance uses by contextual factors.

**Sex**				
	**Alcohol use (self-reported)**			
	**None**	**Non-daily**	**Daily**	**p-value**
**Male**	1 (1.3%)	73 (98.7%)	0 (0%)	
**Female**	3 (3.9%)	74 (96.1%)	0 (0%)	.620
	**Amphetamines use (self-reported)**			
**Male**	71 (95.9%)	3 (4.1%)	0 (0%)	.360
**Female**	76 (98.7%)	1 (1.3%)	0 (0%)	
	**Cannabis use (self-reported)**			
**Male**	55 (74.3%)	14 (19%)	5 (6.7%)	.209
**Female**	65 (84.4%)	7 (9.1%)	5 (6.5%)	
	**Cocaine use (self-reported)**			
**Male**	73 (98.7%)	1 (1.3%)	0 (0%)	.490
**Female**	77 (100%)	0 (0%)	0 (0%)	
	**Gambling (self-reported)**			
**Male**	64 (86.5%)	0 (0%)	10 (13.5%)	1
**Female**	66 (85.7%)	0 (0%)	11 (14.3%)	
**Residence**				
	**Alcohol use (self-reported)**			
**Urban**	3 (2.5%)	114 (97.5%)	0 (0%)	1
**Rural**	1 (3%)	33 (97%)	0 (0%)	
	**Amphetamines use (self-reported)**			
**Urban**	114 (97.5%)	3 (2.5%)	0 (0%)	1
**Rural**	33 (97%)	1 (3%)	0 (0%)	
	**Cannabis use (self-reported)**			
**Urban**	93 (79.5%)	16 (13.7%)	8 (6.8%)	1
**Rural**	27 (79.4%)	5 (14.7%)	2 (5.8%)	
	**Cocaine use (self-reported)**			
**Urban**	116 (99.1%)	1 (0.9%)	0 (0%)	1
**Rural**	34 (100%)	0 (0%)	0 (0%)	
	**Gambling (self-reported)**			
**Urban**	102 (87.1%)	0 (0%)	15 (12.8%)	.57
**Rural**	28 (82.3%)	0 (0%)	6 (17.6%)	
**Mental health follows up**				
	**Alcohol use (self-reported)**			
**No**	4 (2.9%)	135 (97.1%)	0 (0%)	1
**Yes**	0 (0%)	12 (100%)	0 (0%)	
	**Amphetamines use (self-reported)**			
**No**	137 (98.5%)	2 (1.5%)	0 (0%)	**.032**
**Yes**	10 (83.3%)	2 (16.6%)	0 (0%)	
	**Cannabis use (self-reported)**			
**No**	112 (80.7%)	20 (14.4%)	7 (5%)	**.052**
**Yes**	8 (66.7%)	1 (8.3%)	3 (25%)	
	**Cocaine use (self-reported)**			

**No**	139 (100%)	0 (0%)	0 (0%)	.079
**Yes**	11 (91.7%)	1 (8.3%)	0 (0%)	
	**Gambling (self-reported)**			
**No**	121 (87%)	0 (0%)	18 (13%)	.375
**Yes**	9 (75%)	0 (0%)	3 (25%)	

All variables refer to the past 3 months. Parental use variables are adolescent-reported and indicate whether the parent used alcohol or cannabis at least once per month (≥monthly) versus no use. Only alcohol, cannabis, or no drug use were reported.

Table S3. Association between adolescents' reported paternal substance use and adolescents' use patterns.

**Reported father use (as reported by the adolescent participant)**				
	**Participant alcohol use (self-reported)**			
	**None**	**Non-daily**	**Daily**	**p-value**
**Alcohol**	0 (0%)	5 (100%)	0 (0%)	1
**Cannabis**	0 (0%)	10 (100%)	0 (0%)	
**None**	4 (3.0%)	132 (97.0%)	0 (0%)	
	**Participant amphetamines use (self-reported)**			
**Alcohol**	4 (80.0%)	1 (20.0%)	0 (0%)	**<.001**
**Cannabis**	7 (70.0%)	3 (30.0%)	0 (0%)	
**None**	136 (100%)	0 (0%)	0 (0%)	
	**Participant cannabis use (self-reported)**			
**Alcohol**	3 (60%)	1 (20%)	1 (20%)	**.024**
**Cannabis**	6 (60%)	1 (10%)	3 (30%)	
**None**	111 (81.6%)	19 (14%)	6 (4.4%)	
	**Participant cocaine use (self-reported)**			
**Alcohol**	5 (100%)	0 (0%)	0 (0%)	.099
**Cannabis**	9 (90.0%)	1 (10.0%)	0 (0%)	
**None**	136 (100%)	0 (0%)	0 (0%)	
	**Participant gambling (self-reported)**			
**Alcohol**	5 (100%)	0 (0%)	0 (0%)	.685
**Cannabis**	8 (80.0%)	0 (0%)	2 (20.0%)	
**None**	117 (86.0%)	0 (0%)	19 (14.0%)	
**Reported mother use (as reported by the adolescent participant)**				
	**Participant alcohol use**			
**Alcohol**	0 (0%)	2 (100%)	0 (0%)	.197
**Cannabis**	1 (16.7%)	5 (83.3%)	0 (0%)	
**None**	3 (2.1%)	140 (97.9%)	0 (0%)	
	**Participant amphetamines use**			
**Alcohol**	1 (50.0%)	1 (50.0%)	0 (0%)	.059
**Cannabis**	6 (100%)	0 (0%)	0 (0%)	
**None**	140 (97.9%)	3 (2.1%)	0 (0%)	
	**Participant cannabis use**			
**Alcohol**	1 (50.0%)	0 (0%)	1 (50%)	.116
**Cannabis**	4 (66.7%)	1 (16.7%)	1 (16.7%)	
**None**	115 (80.4%)	20 (14.0%)	8 (5.6%)	
	**Participant cocaine use**			
**Alcohol**	2 (100%)	0 (0%)	0 (0%)	1
**Cannabis**	6 (100%)	0 (0%)	0 (0%)	

**None**	142 (99.3%)	1 (0.7%)	0 (0%)	
	**Participant gambling**			
**Alcohol**	2 (100%)	0 (0%)	0 (0%)	
**Cannabis**	4 (66.7%)	0 (0%)	2 (33.3%)	.407
**None**	124 (86.7%)	0 (0%)	19 (13.3%)	

Reported paternal use categories were mutually exclusive: 'Alcohol' indicates any paternal alcohol use and no paternal cannabis use; 'Cannabis' indicates any paternal cannabis use (with or without alcohol); 'None' indicates no reported paternal use of either substance. All variables refer to the past 3 months.
